# 人肺癌A549细胞系肿瘤干细胞的筛选和鉴定

**DOI:** 10.3779/j.issn.1009-3419.2013.08.02

**Published:** 2013-08-20

**Authors:** 晖 夏, 长海 于, 文 张, 宝石 张, 英男 赵, 芳 方

**Affiliations:** 100048 北京，解放军总医院第一附属医院胸心外科 Department of Carciothoracic Surgery, the First Afliated Hospital of the General Hospital of PLA, Beijing 100048, China

**Keywords:** A549细胞, 肿瘤干细胞, 肺肿瘤, A549 cells, Cancer stem cells, Lung cancer

## Abstract

**背景与目的:**

肺癌干细胞是肺癌恶性表型的根源和潜在的治疗靶点，从人肺癌A549细胞株中分离肺癌干细胞，观察特异性干细胞标志物分子的表达，为进一步的干细胞研究提供试验基础。

**方法:**

接种肺癌A549细胞株，经流式细胞术，特异性筛选分离肺癌干细胞，观察克隆形成能力、细胞增殖能力和体外致瘤能力的差别，并分别用RT-PCR和Western blot的方法分析干细胞标志物分子CD133和ABCG2的表达。

**结果:**

经过流式细胞仪成功分选了人肺腺癌A549细胞系SP细胞亚群，结果表明此SP细胞亚群约占A549细胞总数的5.93%，经维拉帕米处理后Hoechest33342阴性/弱阳性细胞含量下降为0.32%，SP细胞克隆形成能力，细胞增殖能力和体外致瘤能力均明显高于非SP细胞。RT-PCR和Western blot结果发现，筛选分离的肺癌SP细胞群高表达干细胞标志物分子CD133和ABCG2。

**结论:**

通过流式细胞术可以筛选分离高表达CD133和ABCG2分子的肺癌干细胞，可用于进一步的研究中。

## Abstract (en)

**Background and objective:**

Lung cancer stem cells are the root causes of lung cancer malignant phenotype and potential therapeutic target, the aim of this study is to isolate and characterize the cancer stem cells in the lung adenoearcinomas cell line A549, so as to provide an experimental basis for further stem cell research.

**Methods:**

The cancer stem cells were isolated from the lung adenoearcinomas cell line A549 using FACS. And the difference of colony formation, cell proliferation and tumorigenicity *in vitro* were also tested. The expression of CD133 and ABCG2 were evaluated by RTPCR and Western blot.

**Results:**

The percentage of SP cells was 5.93% of A549 and 0.32% of A549 afer incubation with verapamil. The results showed that there were signifcantly higher expression of CD133 and ABCG2 on SP cells than that of non-SP cells. And the ability of colony formation, cell proliferation and tumorigenicity in SP cell group were remarkably higher than that in non-SP cell group.

**Conclusion:**

Our results suggested that the cancer stem cells with higher expression of CD133 and ABCG2 can be isolated from the lung adenoearcinomas cell line A549 using FACS and be used in the further research experiments.

非小细胞肺癌(non-small cell lung cancer, NSCLC)约占肺癌总数的80%-85%，对放疗和化疗易产生抵抗性，患者5年的生存率低，生存质量差^[1, 2]^。深入研究肺癌发生，发展的分子机制，探寻新的治疗靶点，提高NSCLC的临床治疗效果是人们关注的问题。肿瘤干细胞(cancer stem cells, CSCs)是处于各种不同分化程度的一小群干细胞样细胞，具有自我更新和无限增殖能力以及多向分化潜能，是肿瘤形成的起始细胞并维持肿瘤的持续生长，在肿瘤的发生、进展、转移、复发及耐药中起关键作用。目前研究^[3, 4]^认为，肿瘤干细胞对常规治疗有抵抗性，且具有再次形成肿瘤的能力，因而其可能是肿瘤复发转移的根源。研究^[5, 6]^表明，CD133阳性细胞具有高度增殖分化潜能和体内成瘤能力及干细胞样特性；ABC转运蛋白家族中ABCG2(ATP-binding cassette superfamily G member 2, ABCG2)与干细胞泵出Hoechst染料的特性有关，经此方法分离得到的SP细胞经鉴定就可确认为干细胞，ABCG2已被认为是一种筛选干细胞的表面标志物分子；为了研究肿瘤干细胞的生物学特性，如何分离出肿瘤干细胞是关键的一步。肺癌干细胞被视为是肺癌恶性表型的根源和潜在的治疗靶点。本研究选用NSCLC细胞系A549为研究对象，通过免疫荧光激活的流式细胞术筛选A549细胞系中SP(side population)细胞亚群，并观察CD133和ABCG2的表达，为探索NSCLC治疗的新途径提供实验依据和理论基础。

## 1 材料与方法

### 1.1 主要仪器和试剂

人肺癌A549细胞株购于军事医学科学院。二甲基亚砜(Dimethyl sulfox ide, DMSO)、Hoechesl33342、维拉帕米(Verapamil)购于Sigma公司；胎牛血清购于Hyclone公司；DMEM(Dulbecco's modifed eagle medium)培养基购于Gibco公司。细胞培养箱为日本Tabai Espec Co.生产。

### 1.2 细胞培养

人肺癌A549细胞，常规培养于含10%灭活胎牛血清的DM EM培养基中，置37 ℃、5%CO_2_孵箱内培养。取对数生长期细胞制备成1×10^6^/L单细胞悬液，加入Hoechst33342染料(5 μg/mL)，37 ℃避光条件下，孵育90 min。对照组细胞中，同时加入维拉帕米(100 mM)，按上述过程处理细胞。各组细胞经FACS缓冲液处理后，在355 nm激发光波长处，应用流式细胞仪检测各组细胞设定参数的变化，测量前向散射和侧向散射参数，以Hoechst red为X轴，Hoechst blue为Y轴作二维散点图，将维拉帕米组缺失的区域设为SP细胞范围，分选出SP干细胞亚群和非SP干细胞亚群，并计算百分比。将分选出的SP细胞和非SP细胞分别接种于10%的软琼脂，观察两组细胞克隆形成能力的差别。MTT法观察所筛选的两组细胞增殖能力的区别，并绘制细胞生长曲线。

### 1.3 RT-PCR分析

经流式细胞仪分选的SP细胞和非SP细胞，以1×10^6^/瓶接种A549细胞于T25培养瓶，置5%CO_2_孵箱。取对数生长中期的细胞，Trizol法提取细胞总R NA，RT-PCR分析干细胞标志物分子CD133和ABCG2 mRNA表达的变化，产物经1%琼脂糖凝胶电泳分离，0.5%溴化乙锭染色后，凝胶成像仪观察图像并计算灰度值，分析各组间差异。

### 1.4 Western blot分析

经流式细胞仪分选的SP细胞和非SP细胞，以1×10^6^/瓶接种A549细胞于T25培养瓶，置5%CO_2_孵箱。取对数生长中期的细胞，加入细胞裂解液，15, 000 rpm、10 min，取上清，Bradford法蛋白定量，经SDS-PAGE凝胶电泳后将蛋白转到硝酸纤维素膜上，5%脱脂奶粉封闭，再分别加入CD133和ABCG2分子的一抗(1:1, 000)和辣根酶标记的二抗(1:500)，室温下振荡孵育1 h，ECL发光试剂盒发光显影，Bio-Rad图像分析软件进行图像扫描和分析。同时用内参蛋白Actin对目的分子进行标准化。

### 1.5 致瘤能力实验

经流式细胞仪分选的上述SP细胞和非SP细胞，以1×10^4^的密度接种至裸鼠皮下，2周后观察两组细胞致瘤能力的差别。

### 1.6 统计学处理

应用SPSS 13.0统计软件进行单因素方差分析，*P* < 0.05表示差别有统计学意义。

## 2 结果

### 2.1 A549细胞中SP干细胞亚群的分选

如图 1所示，肺癌细胞系A549细胞中SP细胞分布情况。图 1A显示SP细胞所占比例为5.93%。图 1B为加入维拉帕米的对照组，SP细胞基本消失为0.32%。图 1C显示SP细胞可以形成明显的肿瘤细胞克隆。图 1D显示非SP细胞不能形成明显的细胞克隆。

**1:**
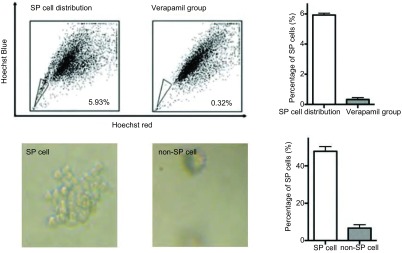
流式细胞术筛选SP干细胞亚群 Isolated slide population (SP) cancer stem cells using FACS

### 2.2 细胞增殖结果

图 2所示，经流式细胞术筛选的SP细胞和非SP细胞在无血清培养条件下，分别绘制生长曲线，两组细胞增殖能力差别明显，SP细胞具有明显的增殖效应(*P* < 0.001, *n*=6)。

**2:**
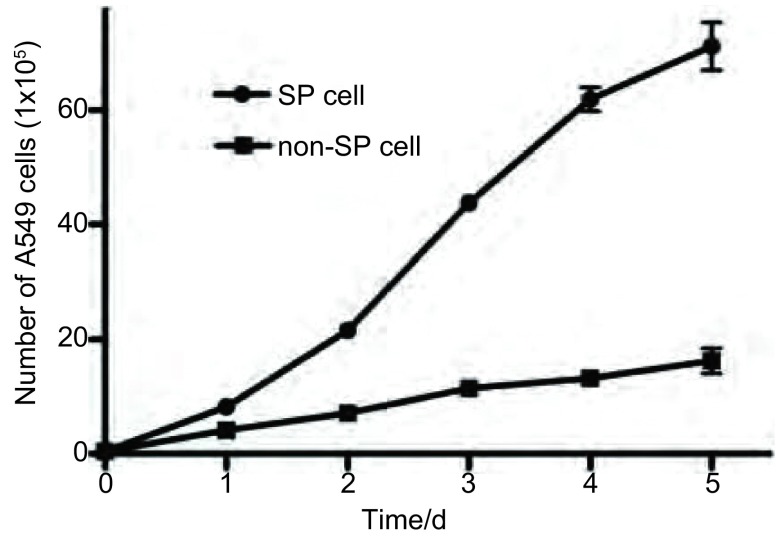
SP细胞和非SP细胞增殖能力比较 The ability of cell proliferation between SP and non-SP cells

### 2.3 RT-PCR结果

图 3结果显示，SP干细胞亚群中CD133和ABCG2 mRNA明显高于非SP干细胞亚群(*P* < 0.05, *n*=5)。

**3:**
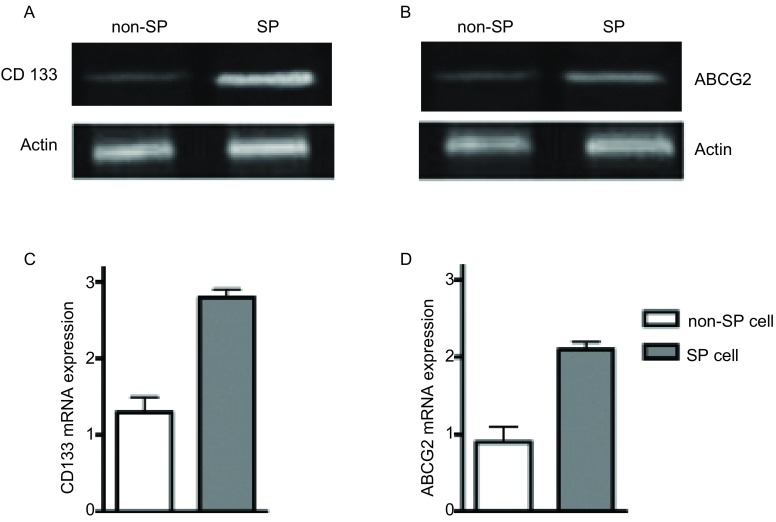
CD133和ABCG2 mRNA表达水平。A、C：CD133 mRNA表达；B、D：ABCG2 mRNA表达。 The expression level of CD133 and ABCG2 mRNA. A, C: CD133 mRNA expression; B, D: ABCG2 mRNA expression.

### 2.4 Western blot结果

图 4结果显示，SP干细胞亚群中CD133和ABCG2 mRNA明显高于非SP干细胞亚群(*P* < 0.05, *n*=5)，各组A549细胞的内参蛋白Actin表达水平一致。

**4:**
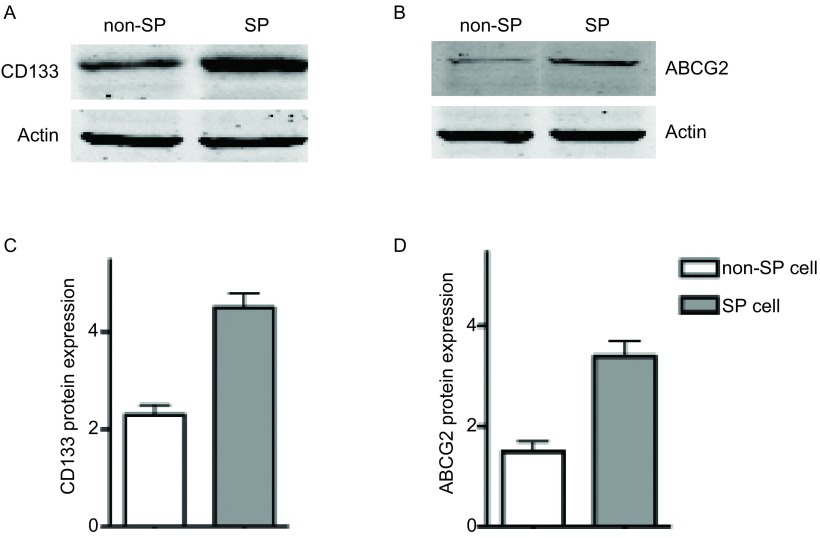
CD133和ABCG2蛋白表达水平。A、C：CD133蛋白表达；B、D：ABCG2蛋白表达。 The expression level of CD133 and ABCG2 protein. A, C: CD133 protein expression; B, D: ABCG2 protein expression.

### 2.5 动物实验结果

图 5结果显示，接种SP亚群细胞的动物，2周后可以形成明显的种植瘤，而对照组非SP亚群细胞种植的动物未见有明显的肿瘤形成(*P* < 0.001, *n*=7)。

**5:**
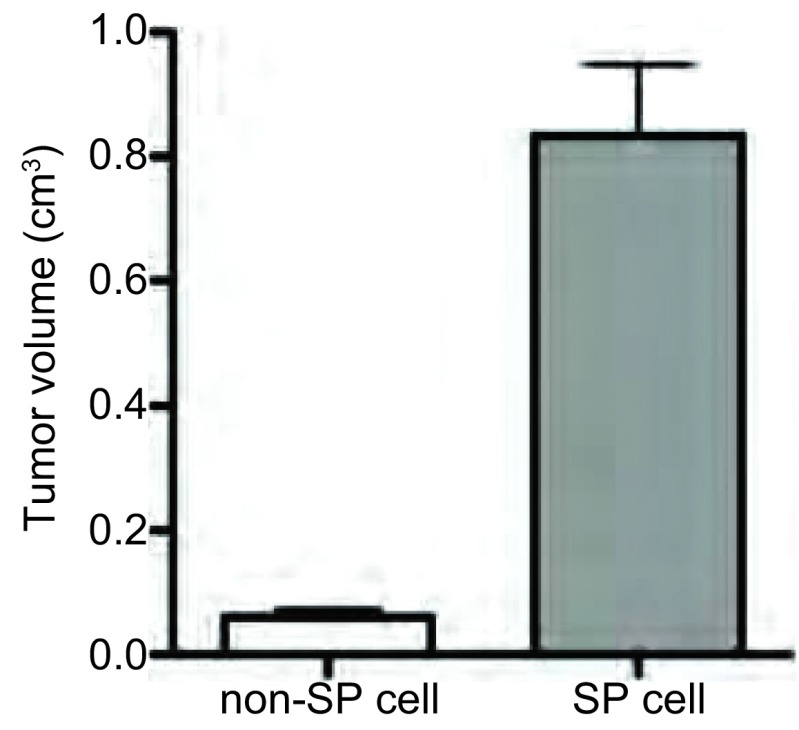
体外致瘤能力实验 The tumorigenicity experiment *in vitro*

## 3 讨论

CSCs也称为"肿瘤起始细胞"(tumor initiating cells, TICs)，具有自我更新和无限增殖能力以及多向分化潜能。研究^[7, 8]^发现，在血液系统、神经系统肿瘤、乳腺癌等肿瘤中，干细胞样细胞决定了肿瘤的发生和发展过程。肿瘤干细胞学说和理论不仅为肿瘤发生、发展和转移机制的研究带来新的思路，同时为肿瘤临床诊断和治疗带来新希望，是肿瘤学领域具有划时代意义的全新理论。本研究中，肺癌干细胞的筛选主要是利用干细胞中转运蛋白可以主动外泵荧光染料(Hoechst染料)的特点，结合流式细胞术，经过双波长分析，激发不同颜色的弱红光和蓝光，此群呈鸟嘴状分布的细胞主要集中于低荧光信号区域，仅占总体肺癌细胞的极少数，即为肺癌的边缘群细胞(SP细胞)，而不具有外排功能的、高荧光信号的细胞即为占细胞整体绝大多数的非SP细胞。

我们的研究结果表明，经过流式细胞仪成功分选了人肺腺癌A 5 49细胞系SP细胞亚群，结果表明此SP细胞亚群约占A 549细胞总数的5.93%，经维拉帕米处理后Hoechest33342阴性/弱阳性细胞含量下降为0.32%，结合文献报道，维拉帕米可以阻断SP细胞排出Hoechest33342，提示此亚群细胞体现Hoechest33342阴性/弱阳性的特性，为筛选所分离的SP细胞，其克隆形成能力明显高于非SP细胞。同时，MTT结果提示，经流式细胞术筛选的SP细胞和非SP细胞在无血清培养条件下，两组细胞增殖能力差别明显，SP细胞具有明显的增殖效应。体外致瘤能力实验发现，接种SP亚群细胞的动物，2周后可以形成明显的种植瘤，而对照组非SP亚群细胞种植的动物未见有明显的肿瘤形成。

据文献报道，CD133为人造血干细胞和祖细胞的标志物分子，同时在多种肿瘤组织中还发现CD133分子的高表达可以作为肝癌、前列腺癌、胰腺癌、结肠癌等肿瘤干细胞的阳性标记物分子。研究^[9, 10]^发现，CD133阳性细胞具有自我更新、不断分化并促进肿瘤生长的特性，与患者的淋巴转移和预后相关。我们的试验结果也表明，经过流式细胞仪筛选的肺癌SP细胞群，明显地高表达干细胞标志物分子CD133，提示经过筛选分离的肺癌SP细胞群具有干细胞样特性。ABCG2分子属于ABC转运蛋白超家族，编码由655个氨基酸组成的跨膜蛋白，在白血病细胞或乳腺癌等肿瘤干细胞表面均有表达。研究^[11, 12]^发现，白血病细胞或乳腺癌细胞的耐药性与ABCG2蛋白密切相关，下调ABCG2分子的表达可以降低肿瘤细胞耐药现象的发生，提示ABCG2作为肿瘤干细胞表面的标志物分子，可能与肿瘤干细胞参与耐药的过程相关。我们的试验结果表明，经过流式细胞仪筛选的肺癌SP细胞群明显地高表达干细胞标志物分子ABCG2。

我们的研究证明：流式细胞术筛选的S P细胞，经mR NA和蛋白水平分析，均高表达CD133和ABCG2；我们所筛选的SP细胞可以形成明显的肿瘤细胞克隆，而非SP细胞不能形成明显的细胞克隆，SP细胞细胞增殖能力和体外致瘤能力均明显高于非SP细胞，上述结果明确提示我们所筛选的细胞为肺癌干细胞。综上所述，本研究中经流式细胞术分离筛选的肺癌SP细胞群，表达特异性的干细胞标志物分子CD133和ABCG2，可以用于进一步的肺癌干细胞的研究中，为肺癌干细胞参与肺癌的发生，发展及耐药等机制的深入研究提供了实验依据和理论基础，具有一定的实际应用价值和意义，但具体相关机制还有待进一步的实验来证实。
